# Electromagnetic Regulation of Electrolyte Solution Heat Convection in Microchannels

**DOI:** 10.3390/mi9060262

**Published:** 2018-05-28

**Authors:** Miaomiao Song, Xiaoting Chi, Yahui Wang, Yu Ma

**Affiliations:** 1 No. 703 Research Institute of China Shipbuilding Industry Corporation, Harbin 150078, China; 18846448849@163.com; 2 School of Energy Science and Engineering, Harbin Institute of Technology, Harbin 150001, China; cxt@stu.hit.edu.cn (X.C.); wangyahui@hit.edu.cn (Y.W.); 3 Sino-French Institute of Nuclear Engineering and Technology, Sun Yat-sen University, Zhuhai 519082, China

**Keywords:** microchannel, convection heat convection, electrolyte solution, electromagnetic regulation

## Abstract

With the rapid development of microelectronics and micro-electromechanical system technology, the electronic components have become smaller and the performance has become higher. Under this condition, however, their energy consumption and heat production have also increased continuously, which poses a great challenge to heat dissipation. In this paper, electromagnetic driving technology is applied to drive the electrolyte solution flow within a microchannel to realize efficient heat convection with microchannel walls. By changing the magnitude and direction of electric–magnetic field, the regulation of heat convection performance is studied. The results show that the *Nu* number of microchannel increases as the *Ha* number and magnetic direction angle increases, while it decreases as the potential difference increases. According to the average index of the four factors, it was determined that the electrolyte solution had the best heat convection performance with *Ha* = 0.05, *Vb* = 0.00006, *Pe* = 90, and *α* = 90°. After that, sensitivity analysis of the *Ha* number, potential difference and magnetic direction angle was used to regulate the heat convection performance. This paper may provide some theoretical support for the design of microelectronics and micro-electromechanical systems.

## 1. Introduction

In recent years, the development of microelectronics and micro-electromechanical systems has undergone much investigation, which has made the electronic components smaller and the performance higher [1]. Under this condition, however, their energy consumption and heat production have also increased continuously [2], which poses a great challenge to heat dissipation. During operation, electronics shows great sensitivity to temperature and excessive temperature will reduce their performance and reliability [3]. Therefore, we must find a reasonable and effective heat dissipation method to control the temperature of electronic devices for better stability and safety during operation. The purpose of developing an electronic component cooling technology is to miniaturize and integrate existing liquid cooling techniques, so that these can be applied to microchannels [4]. To this end, how to drive liquid flow stably in the microchannel without vibration and noise was the focus of the current research.

In order to achieve stable flow in microchannels and narrow microchannels, the flow characteristics and influencing factors need to be studied first. On this issue, many researchers have carried out a lot of valuable research, which provides strong support for later work [5,6,7]. After comparison, the application of magnetohydrodynamics (MHD) may be a good choice. MHD is the phenomenon by which a conductive liquid undergoes a Lorentz force and moves under the action of mutually perpendicular magnetic and electric fields [8]. Recently, the application of MHD has played an important role in many engineering fields, including aerodynamics [9], metallurgy [10], thermonuclear experimental reactors [11], and others [12], owing to its attractive advantages [13], such as high flexibility, low application voltage, great economy, no moving parts, highly driving force, high safety and reliability. Some studies of its application in microfluidics have been made. Parashar et al. [14] and Krause et al. [15] introduced the application of MHD in electromechanical dynamics and liquid metal, respectively. They argued that the MHD effect should be considered in these phenomena. Zhang et al. [16] numerically studied the acceleration/deceleration control of three-dimensional MHD flow in a channel. After numerical simulation, the relationship between the acceleration/deceleration effect and electric–magnetic fields were determined. Jang et al. [17] studied a novel micropump, which pumping mechanism was based upon MHD principles, and the performance of the MHD micropump obtained theoretically in a single phase was compared with the experimental results. Ahmed et al. [18] numerically simulated a direct current (DC) electromagnetic pump for liquid aluminum at a large Reynolds number under an externally-imposed, non-uniform magnetic field. Lim et al. [19] presented a continuous flow MHD micropump with side walled electrodes using Lorentz force, and established a theoretically simplified MHD flow model. To study the combined influences of electro–magneto–hydrodynamic forces in controlling the fluid flow, Chakraborty et al. [20] developed a mathematical model for parallel plate rectangular microchannels. The results shows that the volumetric flow rates substantial increased with a low-magnitude magnetic field but stopped increasing with a high-magnitude magnetic field. Based on the above, the MHD-driven technique can be treated as a stable driving method. However, most of this work focused on the fluid flow in microchannels without considering heat convection. The study of heat convection in microchannels originated from the transport phenomena in porous media. Many works on this issue provide rich experience for the benefit of later research [21]. Yazdi et al. [22] studied MHD liquid flow and heat transfer over a non-linear permeable stretching surface in the presence of chemical reactions and partial slip. The analytical solution in this research may be potentially influential in controlling wall shear stress as well as the local Nusselt and Sherwood numbers. Sarkar et al. [23] analyzed the effect of nanofluids on thermally fully-developed MHD flows through microchannels.

Current studies of MHD-driven convection heat transfer in microchannels mainly focus on nanofluids and metal fluids, and research on the heat convection of electrolytes in microchannels has not yet been reported. To improve this situation, this paper proposes the use of electromagnetic drive to achieve the flow and heat convection of the electrolyte solution in the microchannel. Through dimensionless derivation, the standard parameters of the heat convection for the electrolyte solution under the electromagnetic drive are obtained. The flow of the electrolyte solution in the microchannel and its heat convection control method are studied by numerical simulation.

## 2. Physical Modeling

**Figure 1:**
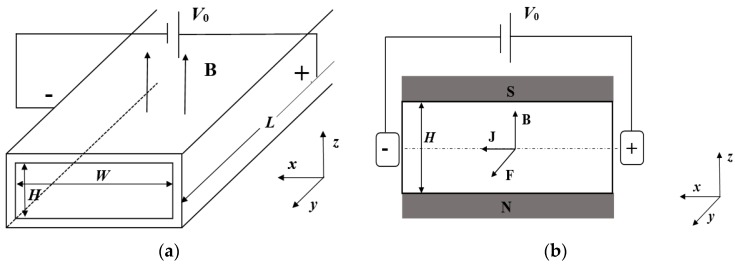
Schematic diagram of a rectangle electromagnetically-driven microchannel. (**a**) Three-dimensional model schematic; (**b**) Two-dimensional schematic diagram of *x*–*z* cross-section.

This section introduces the physical model of the electromagnetic-driven electrolyte solution in a three-dimensional rectangular microchannel, as shown in Figure 1. The length, height and width of the microchannel are denoted by *L*, *H* and *W*, respectively, and the temperature of the walls is denoted by *T_w_*. The microchannel is filled with KCl electrolyte solution with initial temperature *T*_0_ at the inlet, and *T_w_* > *T*_0_. Two electrodes with potential difference *V*_0_ are applied to the left and right surfaces, resulting in a current density **J** (*x*-direction). A magnetic field with strength **B** is applied in *z*-direction. These two fields generate a Lorentz force **J** × **B**, which drives the electrolyte solution to flow along the +*y* direction of the microchannel and heat exchange with the surrounding walls.

Since the thickness of the double layer of the electrolyte solution in the channel (~10 nm) is much smaller than that of the microchannel, the ion distribution of the electrolyte solution can be assumed to be uniform. The electric field distribution can be described by the Poisson equation [1,24,25]:

(1) $$\nabla^{2}V=\nabla \cdot (u\times B),$$

(2) E=−∇V,

where **u** is the flow velocity, and *V* is the electric potential.

According to Ohom’s law, the current density can be expressed as [26]:

(3) J=σ(−∇V+u×B),

where *σ* is the conductivity of the solution; and **u** × **B** represents the induced electromotive force generated by the ions moving in the electric field.

Due to the small geometry and magnetic Reynolds number, the induced magnetic field can be neglected, so that only the external magnetic field needs to be considered. Therefore, the flow process of the incompressible electrolyte solution under the Lorentz force can be described as the continuity and Navier–Stokes equations:

(4) ∇⋅u=0,

(5) $$-\nabla p+\mu \nabla^{2}u+J\times B=0,$$

where **u** is flow velocity; *ρ* and *μ* denote the density and viscosity of the fluid, and *p* denotes the pressure.

The energy conservation of the electrolyte solution under electromagnetic drive is described by

(6) $$\rho C_{p}u\cdot \nabla T=\lambda \nabla^{2}T+\frac{J\cdot J}{\sigma },$$

where *T* is the temperature of the fluid; *Cp* is the specific heat capacity; *λ* is the thermal conductivity of the electrolyte solution; J⋅J/σ represents the Joule heat source term [27].

To analyze the model mechanism and not be affected by the dimensions, non-dimensionalization is necessary. The dimensionless scaling parameters are defined as follows: dimensionless length:

(7) $$x^{*}=\frac{x}{H},\;y^{*}=\frac{y}{H},\;z^{*}=\frac{z}{H},$$

dimensionless magnetic induction strength:

(8) $$B^{*}=\frac{B}{B_{0}},$$

dimensionless electric potential:

(9) $$V^{*}=\frac{V}{V_{0}},$$

dimensionless current density:

(10) $$J^{*}=J/\left(\sigma V_{0}/W\right),$$

dimensionless speed:

(11) $$u^{*}=\frac{u}{u_{0}},$$

dimensionless pressure:

(12) $$p^{*}=\frac{p}{p_{0}},$$

dimensionless time:

(13) $$t^{*}=\frac{t}{t_{0}},$$

dimensionless temperature:

(14) $$T^{*}=\frac{T}{T_{0}},$$

in which **B**_0_ is the maximal field strength; *V*_0_ is the maximal potential applied to the electrolyte; and **u**_0,_ *p*_0,_ *t*_0_, and *T*_0_ are defined as follows:

(15) $$u_{0}=\frac{\sigma V_{0}B_{0}H^{2}}{\mu W},\;p_{0}=\frac{\mu u_{0}}{H},\;t_{0}=\frac{H}{u_{0}},\;T_{0}=\frac{{\left(\sigma V_{0}/W\right)}^{2}H}{\sigma \rho C_{p}}.$$

Through non-dimensionalization, Equations (1), (3), (5) and (6) can be rewritten as:

(16) $$\nabla^{*}{}^{2}V^{*}=\frac{u_{0}B_{0}H}{V_{0}}\nabla^{*}\cdot \left(u^{*}\times B^{*}\right),$$

(17) $$J^{*}=-\varsigma \;\nabla^{*}V^{*}+Ha^{2}u^{*}\times B^{*},$$

(18) $$-\nabla^{*}p^{*}+\nabla^{*}{}^{2}u^{*}+J^{*}\times B^{*}=0,$$

(19) $$u^{*}\cdot \nabla^{*}T^{*}=\frac{1}{Pe}\nabla^{*}{}^{2}T^{*}+\frac{J^{*}\cdot J^{*}}{\sigma },$$

where ς=H/W is the aspect ratio of the microchannel; $Pe=\rho C_{p}u_{0}H/\lambda$ is the Berkeley number, which characterizes the relative strength of convection and diffusion; $Ha=B_{0}H\sqrt{\sigma /\mu }$ is the Hartmann number that characterizes the ratio of electromagnetic force and viscous force; and $Vb=\frac{u_{0}B_{0}H}{V_{0}}$ is defined as the characteristic number of the electric field, characterizing the ratio of the electromagnetic force and the electric field force.

## 3. Results and Discussion

### 3.1. Model Validation

To verify the present numerical model, an infinitely long rectangular microchannel [26] is tested. When the electric and magnetic fields are applied to the four walls, respectively, satisfying *H* << *W* << *L*, as shown in Figure 1, the velocity of the fully developed flow is isothermally expressed as:

(20) $$u_{x}(z)=-Ha^{-2}\left(\varsigma \frac{dV}{dy}+\frac{dp}{dx}\right)\left[1-\frac{\cosh\left(Haz\right)}{\cosh\left(\frac{Ha}{2}\right)}\right],$$

in which $\frac{dV}{dy}=-E=const$ denotes the electric field intensity in the *y*-direction.

In order to verify the reliability of this model, the velocity distribution of three-dimensional isothermal fully developed flow for the electrolyte solution is calculated firstly under the electromagnetic driving condition.

**Figure 2:**
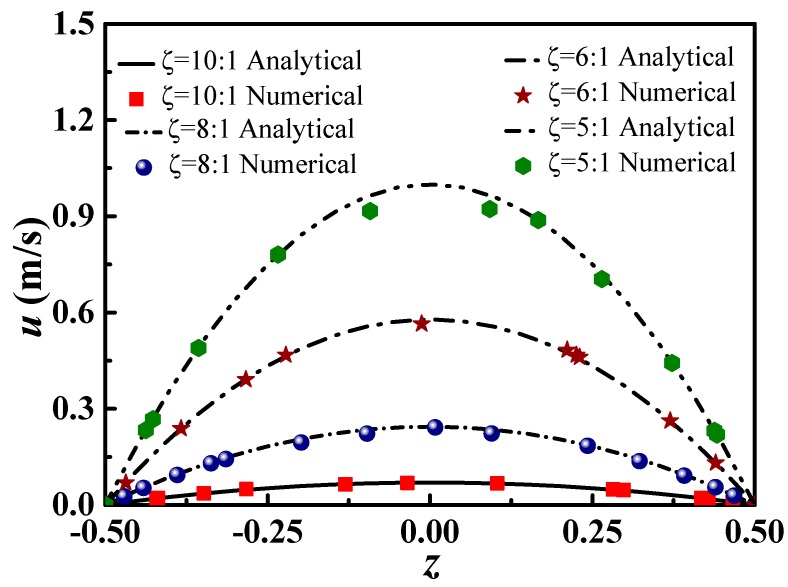
The speed of the middle section along *z*-direction at different aspect ratios.

The KCl electrolyte solution with a concentration of 100 mM is adopted as the working liquid with *σ =* 10 (mΩ)^−1^, *μ =* 0.001 Pa·s, **B** = 0.4 T and *H =* 100 μm. Figure 2 shows the numerical solutions of the velocity distributions along the middle section (W/2), which are compared with the analytical solutions obtained from Equation (20).

As shown in Figure 2, the numerical solutions agree well with the analytical solutions with a large aspect ratio, indicating that the purposed model is accurate and reliable. In considering the small aspect ratio, the numerical solutions are slightly different from the analytical solutions, indicating that the analytical model cannot accurately describe the flow process in the electromagnetically-driven microchannel, and strict three-dimensional numerical simulation methods should be adopted.

### 3.2. Microchannel Heat Convection

#### 3.2.1. Temperature Distribution in the Microchannel

**Figure 3:**
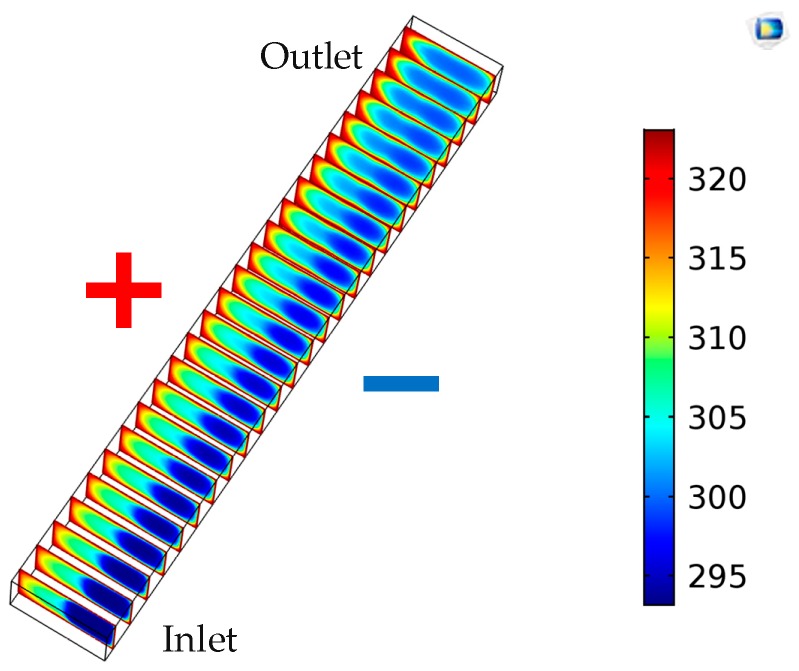
Temperature distribution of various *x*–*z* planes along the *y*-direction.

**Figure 4:**
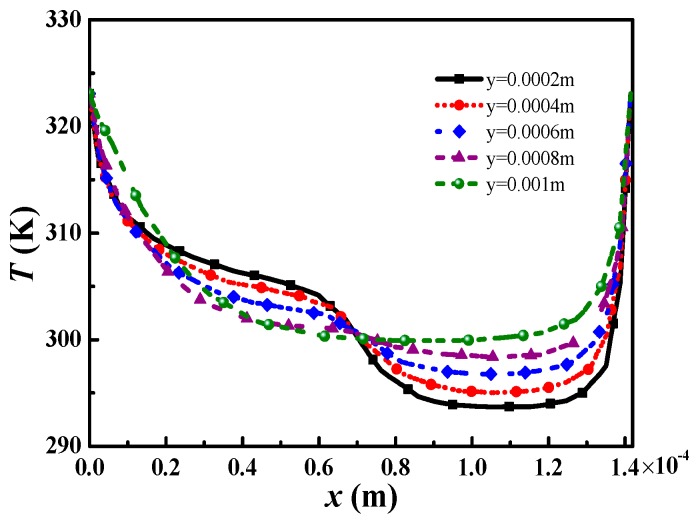
Temperature distribution on the midline of different cross sections along the *y*-direction.

Figure 3 and Figure 4 show the temperature distribution in the microchannel under the electromagnetically-driven system, with boundary temperature *T_w_ =* 323.15 K and initial temperature *T*_0_ = 293.15 K. The aspect ratio of the microchannel is 2. The electrolyte solution flows through the microchannel to achieve heat convection, and its temperature significantly increases at the outlet. Figure 4 shows the temperature distribution along the midline of the *x*–*z* plane at a different *y*-direction position. As shown in Figure 4, as the fluid flow and the temperature distribution of the electrolyte solution becomes flat, and the temperature near the high-potential wall is obviously higher than that of low-potential wall. Therefore, it can be concluded that the heat convective of the electromagnetically-driven electrolyte solution is better on the high-potential wall than low-potential wall. Since the induced electromotive force generated by fluid flow introduces a stronger velocity near the high-potential wall, the heat convection is more intense, and the temperature of the electrolyte solution near the high-potential wall increase significantly.

#### 3.2.2. Influence of Magnetic Strength on Microchannel Heat Convection

The *Nu* number and *Ha* number represent the heat convection ability and the magnetic strength, respectively. To study their relationship, the *Nu* number of the electromagnetically-driven electrolyte solution was calculated at different *Ha* numbers, in which the sodium chloride solution was adopted as the coolant. The inlet temperature was set to 293.15 K, and the microchannel wall temperatures were set to 323.15 K, and were constant during the whole simulation.

**Figure 5:**
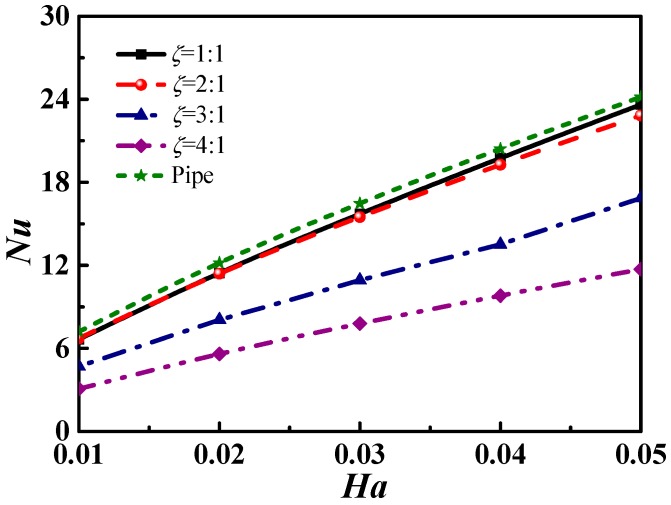
The *Nu* number varies with *Ha* number in microchannels with different cross-sectional shapes.

Figure 5 demonstrates an increase in the *Nu* number with an increase in the *Ha* number regardless of the shape or aspect ratio. The latter increase results in increases in the electromagnetic driving force and the consequent flow speed of the solution, enhancing the heat convection characterized by the *Nu* number. For a certain *Ha* number, the *Nu* number increases as the aspect ratio decreases. Equation (18) shows that the current density increases as the aspect ratio decreases, thus a larger Lorentz force will be produced to enhance heat convection. Figure 5 also shows that the heat convection of the circular microchannel was more intensive than that of square microchannel. This is because in the case of the same cross-section perimeter, the area of the circular cross-section is larger than that of the rectangular cross-section, which led to more electrolytic solution flows and stronger heat convection.

#### 3.2.3. Influence of Magnetic Direction on Microchannel Heat Convection

For certain microchannels and magnetic fields, an effective way to change the *Nu* number is to reset the angular direction of the magnetic field. To this end, in this part, the influence of the magnetic direction on the *Nu* number will be analyzed and discussed.

**Figure 6:**
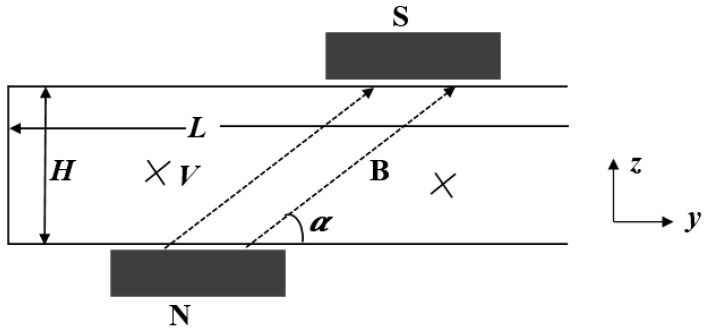
Two-dimensional cross-section of a microchannel that changes the magnetic field angle.

As shown in Figure 6, the angle *α* between the magnetic field and the axis of the microchannel is defined as the magnetic direction angle. The Lorentz force applied to the charged ions changes along with changes in the direction angle *α*.

Under this condition, the momentum equation is modified as [28]:

(21) $$\frac{\partial \rho u_{y}}{\partial t}+\nabla \cdot (\rho uu_{y})=-\frac{\partial p}{\partial y}+\nabla \cdot (\mu \nabla \cdot u_{y})+F_{y},$$

(22) $$\frac{\partial \rho u_{z}}{\partial t}+\nabla \cdot (\rho uu_{z})=-\frac{\partial p}{\partial z}+\nabla \cdot (\mu \nabla \cdot u_{z})+F_{z},$$

in which **u** satisfies $u=j\cdot u_{y}+k\cdot u_{z}$; and *F_y_* and *F_z_* represent the magnetic field volume forces along *y*- and *z*-directions, which can be expressed as:

(23) $$F_{y}=\sigma u_{y}{\left|B\sin\alpha \right|}^{2},$$

(24) $$F_{z}=\sigma u_{z}{\left|B\cos\alpha \right|}^{2},$$

**Figure 7:**
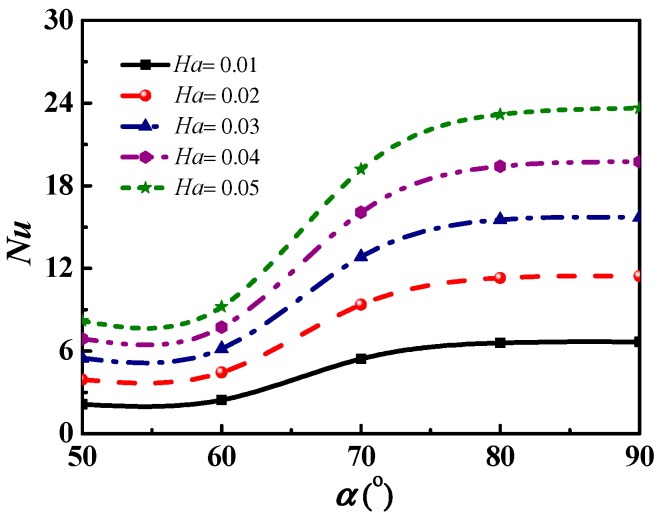
The *Nu* number varies with the magnetic direction in microchannels with an aspect ratio 3:1.

Figure 7 shows the changes in the *Nu* number along with angle *α* increasing with different *Ha* numbers. The aspect ratio of the square microchannel was set to 3:1. The figure shows that the *Nu* number increases as the angle *α* increases. The *Nu* number reaches its maximum at *α =* 90°, since under this direction the induced Lorentz force is parallel to the microchannel axis, and the velocity of the solution reaches the maximum [26].

Figure 7 demonstrates that an increase in *Ha* number will enhance the regulation effect of the magnetic direction. The Lorentz force is more significantly affected by the angle *α* with a large *Ha* number. Besides, when the angle *α* is 60°~80°, the *Nu* number varies most obviously due to the significant change of the sine value and the volumetric force.

#### 3.2.4. Influence of Potential on Microchannel Heat Convection

**Figure 8:**
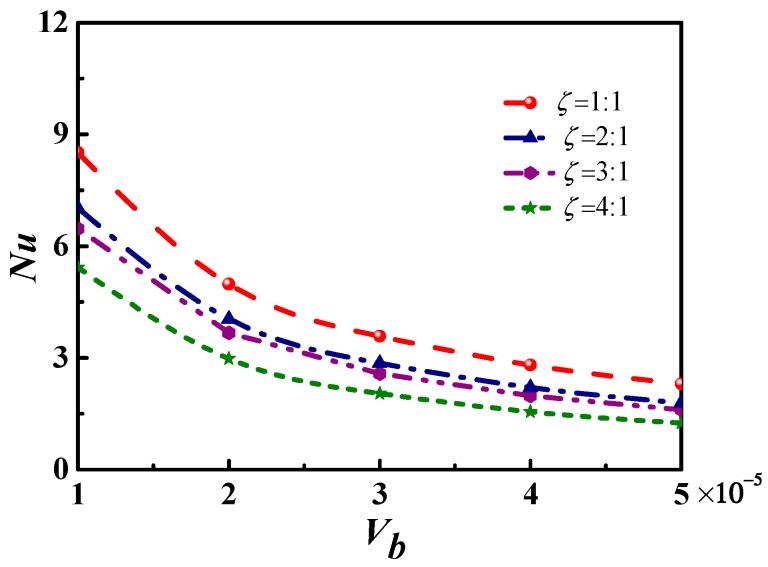
The *Nu* number changes with the *Vb* in the microchannel with different cross-sections, while the wall temperature is 323.15 K.

For a certain microchannel, the heat convection can be regulated by adjusting the potential difference between two electrodes. Figure 8 shows that the *Nu* number decreases as the *Vb* increases, due to the inverse proportion between the *Vb* number and the potential difference. Therefore, the increase in *Vb* will reduce the potential difference and the Lorentz force of the ion.

### 3.3. Impact Degree Analysis by the Orthogonal Experimental Method

#### 3.3.1. Design of the Orthogonal Experiment

As can be seen from the previous discussion, the heat convection performance of the electromagnetically-driven electrolyte solution microchannel will be mainly influenced by the *Ha* number, *Vb* number, angle *α*, and *Pe* number. In this part, a set of orthogonal experiments is designed to analyze the impact degree of these four factors. Five levels were selected for each factor, as shown in Table 1.

Using the orthogonal experimental method, 25 groups of calculation parameters were established to compare the impact degree of the four factors. The results for the *Nu* number are listed in Table 2, and their influences on heat convection were determined based on the numerical results. The data is discussed in the following section.

#### 3.3.2. Range Analysis

The average values and range analyses for the orthogonal experimental results for the 25 groups are shown in Table 3, in which the average values of the four influencing factors including *A* (*Ha* number), *B* (*Vb* number), *C* (*Pe* number), and *D* (angle *α*) are calculated. *A*_5_, *B*_2_, *C*_5_, and *D*_5_ are the priority levels of the four factors, respectively. The order of these elements according to the results is *A* > *D* > *B* > *C*. The range of *A* is the largest, indicating that the *Ha* number has the greatest effect on the heat convection. According to the index average value of these four factors, one can find that the best possible combination is *A*_5_*-B*_2_*-C*_5_*-D*_5_, namely the electrolyte solution in the microchannel. The best heat convection performance exists when *Ha* = 0.05, *Vb* = 0.00006, *Pe* = 90 and *α* = 90° is satisfied.

### 3.4. Sensitivity Analysis of Microchannel Heat Convection Performance

To quantitatively regulate the heat convection of the microchannel, a sensitivity analysis was made for the *Ha* number, *Vb* number and angle *α*.

The sensitivity coefficient is defined as the ratio of the relative rate of change of the objective function to that of the uncertain influence factors, whose expression is written as follows [28]:

(25) $$E=\frac{\Delta A/A}{\Delta F/F},$$

where *A* denotes the objective function, *F* denotes the uncertain influence factor, and *E* denotes the sensitivity coefficient of the objective function *A* to the uncertain influence factor *F*. The larger *E* becomes, the greater the sensitivity coefficient corresponding to the uncertain influence factor.

#### 3.4.1. Sensitivity Analysis of *Ha* Number

**Figure 9:**
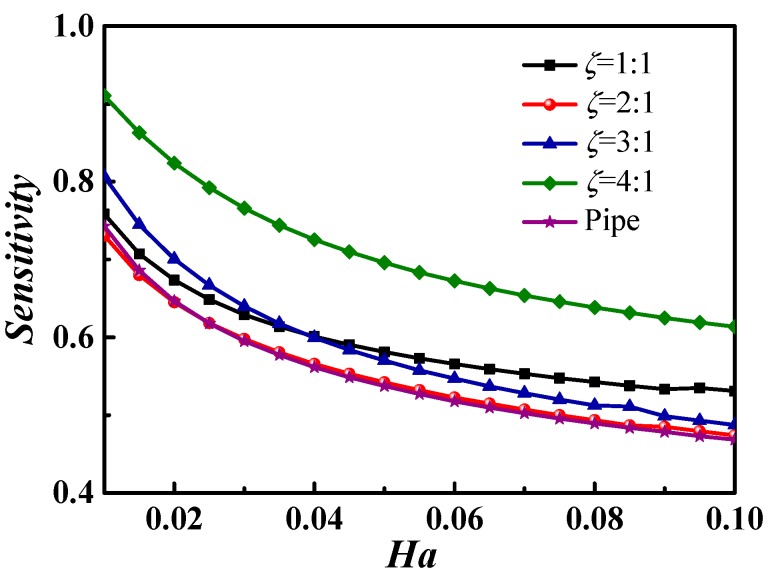
Sensitivity of the *Ha* number to the *Nu* number with various cross-sections.

Firstly, the sensitivity of the *Ha* number on the *Nu* number was analyzed when the magnetic direction was perpendicular to the axis of the microchannel; the results are shown in Figure 9.

Figure 9 illustrates decreases in both the sensitivity coefficient of the *Nu* and the decline rate of the sensitivity with an increase in the *Ha*, when the *Ha* ranges from 0.01 to 0.1, indicating a stronger regulating ability of the *Ha* at a smaller *Ha* number. Figure 9 also shows that the sensitivity of the aspect ratio 4:1 was obviously larger than that of the other square microchannels, and the sensitivity of the circle microchannel (denoted by pipe) was the lowest.

#### 3.4.2. Sensitivity Analysis of *Vb*

**Figure 10:**
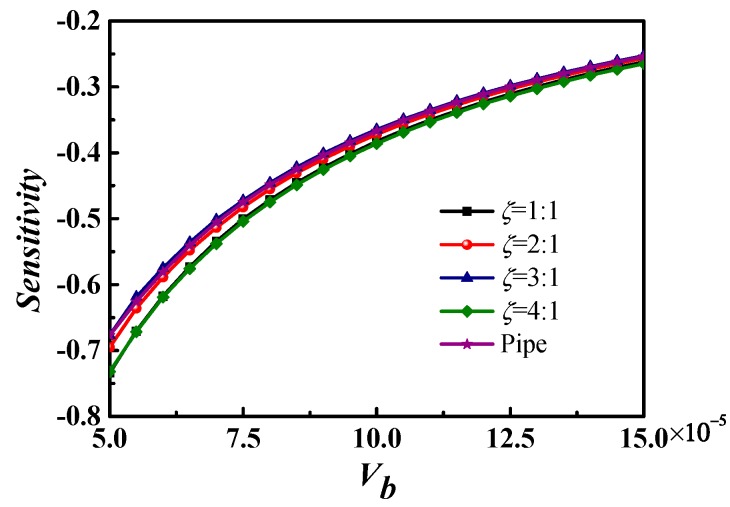
Sensitivity of the *Vb* number to the *Nu* number with various cross-sections.

This part studies the sensitivity of the *Vb* number on the *Nu* number. Figure 10 demonstrates that the sensitivity of the *Vb* number is negative, since the *Vb* number is inversely proportional to the potential difference. The sensitivity decreases as the *Vb* number increases, meaning that the regulating ability of the *Vb* number on *Nu* is stronger at a smaller *Vb* number, and weaker at a larger *Vb* number. The sensitivity coefficients with different aspect ratios show that the aspect ratio has a small impact on the sensitivity coefficient. The sensitivity of the aspect ratio 1:1 and 4:1 was slightly larger than that of other microchannels.

#### 3.4.3. Sensitivity Analysis of the Magnetic Direction

**Figure 11:**
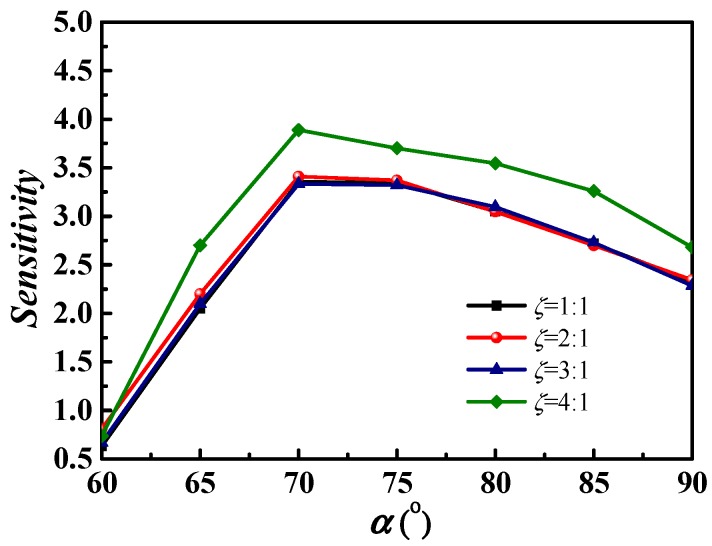
Sensitivity coefficient of magnetic direction on the *Nu* number with various cross-sections.

At last, the sensitivity of the magnetic direction angle *α* on the *Nu* number is analyzed; the results are shown in Figure 11. When the angle *α* varies from 60° to 90°, the sensitivity coefficient of the *Nu* number increases obviously as the angle *α* increases, and reaches maximum at *α =* 70°. After that, the sensitivity coefficient decreases slowly. Therefore, the magnetic direction angle has the strongest adjustment ability to the *Nu* number at about 70°, and the larger direction angle (over 70°) has a stronger adjustment ability than smaller angles (below 70°). The sensitivity coefficient of different aspect ratios shows that the regulating ability of the magnetic direction on the *Nu* number with an aspect ratio of 4:1 is obviously better than that of other aspect ratios.

## 4. Conclusions

The development of microelectronics and micro-electromechanical system technology has produced a great challenge in terms of heat dissipation. To improve this situation, this paper systematically studied the heat convection of microchannels cooled by an electromagnetic driving electrolyte solution. By applying the electric and magnetic fields, the charged ions were driven by Lorentz force along the microchannel, so that efficient heat convection within the microchannel is achieved. For a certain microchannel, the performance of heat convection can be regulated by changing the strength and direction of the magnetic field. The potential difference between electrodes also can provide effective regulation. After the numerical simulation of a microchannel, the following conclusions were drawn:

1. The heat convection of an electromagnetically-driving electrolyte solution microchannel is mainly influenced by the dimensionless characteristic parameters of *Ha* and *Vb* numbers. The *Nu* number increases as the *Ha* number and magnetic direction angle increases, and decreases as the *Vb* number increases.
2. After orthogonal experiments and range analysis, the degree of influence of the main factors of the heat convection in the microchannel is determined as follows: the *Ha* number, the magnetic direction angle, the *Vb* number, and the *Pe* number.
3. After sensitivity analysis, the regulation abilities of the main factors on the heat convection of the microchannel are determined. The first factor is the *Ha* number, which, if it is small enough, has a regulating ability on large *Nu* numbers. Similarly, the *Vb* number shows a strong regulating ability when the *Vb* number is small enough. The magnetic field has the strongest regulating ability when its direction angle is around 70°.

## Figures and Tables

**Table 1:** Parameter table of four factors with five levels.

No	*A* *Ha*	*B* *Vb*	*C* *Pe*	*D* *α/o*
1	0.01	0.00005	50	50
2	0.02	0.00006	60	60
3	0.03	0.00007	70	70
4	0.04	0.00008	80	80
5	0.05	0.00009	90	90

**Table 2:** Orthogonal design table and results for the four factors with five levels.

No	*A* *Ha*	*B* *Vb*	*C* *Pe*	*D* *α/o*	*Nu*
1	0.01	0.00005	50	50	1.06572
2	0.01	0.00006	60	60	1.23486
3	0.01	0.00007	70	70	2.94283
4	0.01	0.00008	80	80	3.64544
5	0.01	0.00009	90	90	3.67242
6	0.02	0.00005	60	70	10.48453
7	0.02	0.00006	70	80	12.36321
8	0.02	0.00007	80	90	11.29287
9	0.02	0.00008	90	50	4.20961
10	0.02	0.00009	50	60	2.6126
11	0.03	0.00005	70	90	27.48634
12	0.03	0.00006	80	50	9.16169
13	0.03	0.00007	90	60	10.00211
14	0.03	0.00008	50	70	11.859
15	0.03	0.00009	60	80	15.03036
16	0.04	0.00005	80	60	8.98464
17	0.04	0.00006	90	70	38.16797
18	0.04	0.00007	50	80	24.98374
19	0.04	0.00008	60	90	26.42209
20	0.04	0.00009	70	50	9.42444
21	0.05	0.00005	90	80	78.24366
22	0.05	0.00006	50	90	42.04679
23	0.05	0.00007	60	50	14.3471
24	0.05	0.00008	70	60	16.38925
25	0.05	0.00009	80	70	35.78468

**Table 3:** Index average and range analysis table of heat convection performance in the microchannel.

Factors	*A*	*B*	*C*	*D*
*K*_1_	12.56127	57.44563	65.17455	38.20856
*K*_2_	41.99269	102.97452	67.51894	45.26918
*K*_3_	73.53946	64.59852	68.60603	99.23901
*K*_4_	107.98288	62.52539	70.73961	134.2664
*K*_5_	186.81148	66.5245	84.29577	136.95034
$\bar{K_{1}}$	2.512254	11.489126	13.03491	7.641712
$\bar{K_{2}}$	8.398538	20.594904	13.503788	9.053836
$\bar{K_{3}}$	14.707892	12.919704	13.721206	19.847802
$\bar{K_{4}}$	21.596576	12.505078	14.147922	26.853282
$\bar{K_{5}}$	37.362296	13.3049	16.859154	27.390068
Priority level	*A*_5_	*B*_2_	*C*_5_	*D*_5_
*Ri*	34.850042	9.105778	3.824244	19.748356
Sequence	*A > D > B > C*			
